# Overexpression of an isopentenyl diphosphate isomerase gene to enhance *trans*-polyisoprene production in *Eucommia ulmoides* Oliver

**DOI:** 10.1186/1472-6750-12-78

**Published:** 2012-10-30

**Authors:** Ren Chen, Yoko Harada, Takeshi Bamba, Yoshihisa Nakazawa, Koichiro Gyokusen

**Affiliations:** 1 Technical Research Institute, Hitachi Zosen Corporation, Osaka, 551-0022, Japan; 2 Department of Biotechnology, Graduate School of Engineering, Osaka University, Suita, Osaka, 565-0871, Japan; 3 Department of Forest and Forest Products Sciences, Faculty of Agriculture, Kyushu University, Fukuoka, 812-8581, Japan

**Keywords:** Isopentenyl diphosphate isomerase, *Trans*-polyisoprene, Natural rubber, Genetic transformation, *Eucommia ulmoides*

## Abstract

**Background:**

Natural rubber produced by plants, known as polyisoprene, is the most widely used isoprenoid polymer. Plant polyisoprenes can be classified into two types; *cis*-polyisoprene and *trans*-polyisoprene, depending on the type of polymerization of the isoprene unit. More than 2000 species of higher plants produce latex consisting of *cis*-polyisoprene. *Hevea brasiliensis* (rubber tree) produces *cis*-polyisoprene, and is the key source of commercial rubber. In contrast, relatively few plant species produce *trans*-polyisoprene. Currently, *trans*-polyisoprene is mainly produced synthetically, and no plant species is used for its commercial production.

**Results:**

To develop a plant-based system suitable for large-scale production of *trans*-polyisoprene, we selected a *trans*-polyisoprene-producing plant, *Eucommia ulmoides* Oliver, as the target for genetic transformation. A full-length cDNA (designated as *EuIPI*, Accession No. AB041629) encoding isopentenyl diphosphate isomerase (IPI) was isolated from *E. ulmoides*. *EuIPI* consisted of 1028 bp with a 675-bp open reading frame encoding a protein with 224 amino acid residues. EuIPI shared high identity with other plant IPIs, and the recombinant protein expressed in *Escherichia coli* showed IPI enzymatic activity *in vitro*. *EuIPI* was introduced into *E. ulmoides* via *Agrobacterium*-mediated transformation. Transgenic lines of *E. ulmoides* overexpressing *EuIPI* showed increased *EuIPI* expression (up to 19-fold that of the wild-type) and a 3- to 4-fold increase in the total content of *trans*-polyisoprenes, compared with the wild-type (non-transgenic root line) control.

**Conclusions:**

Increasing the expression level of *EuIPI* by overexpression increased accumulation of *trans*-polyisoprenes in transgenic *E. ulmoides*. IPI catalyzes the conversion of isopentenyl diphosphate to its highly electrophilic isomer, dimethylallyl diphosphate, which is the first step in the biosynthesis of all isoprenoids, including polyisoprene. Our results demonstrated that regulation of *IPI* expression is a key target for efficient production of *trans*-polyisoprene in *E. ulmoides*.

## Background

**Figure 1:**
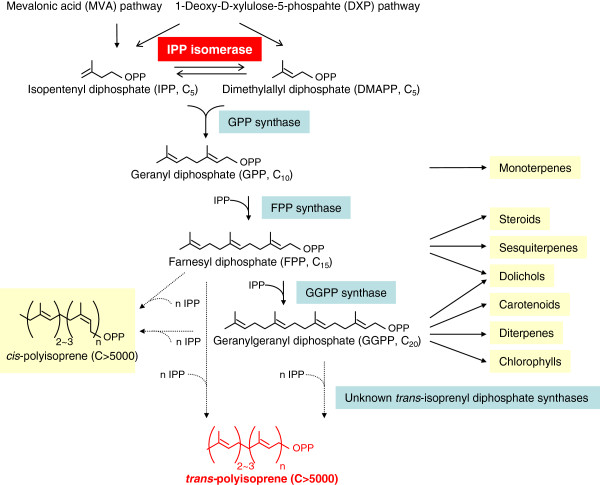
**Overview of *trans*-polyisoprene biosynthesis pathway in plants. **In the isoprenoid biosynthetic pathway, the basic five-carbon unit IPP is synthesized in the cytoplasm via the MVA pathway or in plastids via the DXP pathway. Then, IPP is interconverted to its highly electrophilic isomer, DMAPP, by the isomerase IPI at the first step. DMAPP then loses its inorganic pyrophosphate to form isoprene, which sequentially condenses with IPP to generate the short-chain isoprenoid precursors GPP, FPP, and GGPP. These precursors are further metabolized for the biosynthesis of distinct sets of isoprenoids, such as monoterpenes (C10), sesquiterpenes (C15), diterpenes (C20), and polyisoprenes (C>5000) by various isoprenyl diphosphate synthases.

Natural rubber produced by plants, known as polyisoprene, is the most widely used isoprenoid polymer. Plant polyisoprenes can be classified into two types; *cis*- or (*Z*)-polyisoprene and *trans*- or (*E*)-polyisoprene, depending on the type of polymerization of the isoprene unit (Figure 1) [1,2]. More than 2000 species of higher plants produce latex consisting of *cis*-polyisoprene [3]. *Hevea brasiliensis* (rubber tree), which produces *cis*-polyisoprene, is the key source of commercial rubber because of its high rubber yields, and the excellent physical properties of the rubber products [1]. Relatively few plant species produce *trans*-polyisoprene. Those that are able to produce *trans*-polyisoprene include *Mimusops balata* (Balata), *Palaquium gutta* (Gutta percha) [4,5] and *Eucommia ulmoides*[6-8]. *Trans*-polyisoprene has several specific properties that differ from those of *cis*-polyisoprene. It is more rigid, provides excellent insulation, has an extremely low coefficient of thermal expansion/contraction, and is resistant to acid and alkali. These properties could be exploited in the manufacture of insulated cables, moulds, sports goods, and medical or scientific instruments [9,10]. Currently, *trans*-polyisoprene is mainly produced synthetically, and no plant-based systems have been developed for commercial use.

To develop a suitable plant-based system for the large-scale production of *trans*-polyisoprene, we selected a *trans*-polyisoprene-producing plant, *E. ulmoides* Oliver, as the target for genetic transformation. *E. ulmoides* is a deciduous, dioecious woody plant that produces a *trans*-polyisoprene known as Eu-rubber in the leaves, root, bark, and pericarp [7,8]. In this study, we isolated and overexpressed an isopentenyl diphosphate isomerase (*IPI*) gene in *E. ulmoides* to enhance its *trans*-polyisoprene production. IPI catalyzes the interconversion of isopentenyl diphosphate (IPP) to its highly electrophilic isomer, dimethylallyl diphosphate (DMAPP), which is an essential starter moiety for the first step in biosynthesis of all isoprenoids including polyisoprene (Figure 1). Previous studies reported that overexpression of the *IPI* gene caused accumulation of many related downstream isoprenoid metabolites, such as carotenoids and terpenoid indole alkaloids [11,12]. All these reports suggested that IPI may be a target enzyme for regulating polyisoprene biosynthesis.

## Results

### Cloning and characterization of *EuIPI* cDNA

**Figure 2:**
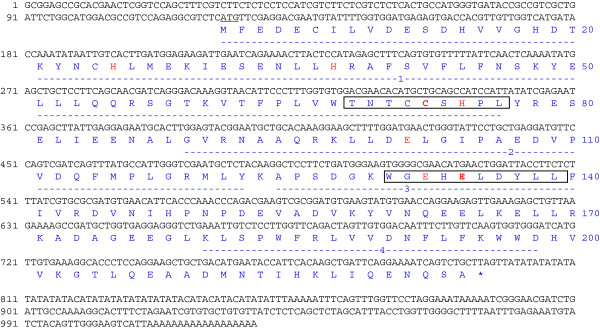
**Nucleotide and deduced amino acid sequences of *EuIPI*. ***EuIPI* (Accession No. AB041629) consists of 1028 bp with a 675-bp ORF encoding a protein with 224 amino acid residues. Numbers of nucleotide sequence and amino acid sequence are indicated on left and right, respectively. Start codon (ATG) is underlined; stop codon (TAG) is marked with *. Broken lines indicate four highly conserved regions of IPIs. Residues critical for catalytic activity of the enzyme are highlighted in red bold font. Boxes show the conserved C (cysteine) motif and the conserved E (glutamic acid) motif.

To isolate the *IPI* gene from *E. ulmoides*, we designed one pair of degenerate primers for RT-PCR based on the sequence of a conserved region in the known plant IPIs, including *Arabidopsis thaliana* IPI (U47324), *Bupleurum chinense* clone IPI (GQ433719), *Camptotheca acuminata* IPI1 (AF031079), *H. brasiliensis* IPI1 (AB294696); *Ipomoea* sp. Kenyan IPI (AB499048), *Nicotiana tabacum* IPI1 (AB049815), *Periploca sepium* IPI (AB091677), *Pinus taeda* IPI (GQ476784), *Solanum lycopersicum* IPI (EU253957), and *Zea mays* IPI (AF330034). The amplified PCR product (228 bp) was used as a probe to screen the cDNA library constructed from a mature *E*. *ulmoides* tree. One positive clone carried a full-length cDNA insert that showed the highest homology to known IPIs. This sequence was designated as *EuIPI* (Accession No. AB041629). Sequence analysis showed that *EuIPI* was 1028 bp in length, and contained a 675-bp open reading frame (ORF). The ORF encoded a protein of 224 amino acid residues with a predicted molecular mass of 25.96 kDa and an isoelectric point of 4.68 (Figure 2). Sequence alignment of the deduced amino acid sequence against those of other IPIs revealed that EuIPI showed high identity (84–92.7%) to other plant IPIs. Similar to other eukaryotic IPIs reported previously, EuIPI contained a number of highly conserved regions that are common to IPIs from higher plants and humans to yeasts [11,13], and two residues, C (cysteine) and E (glutamic acid), in the TNTCCSHPL motif and the WGEHEXDY motif (Figure 2), respectively, which are critical for catalytic activity of the enzyme [13-16].

**Figure 3:**
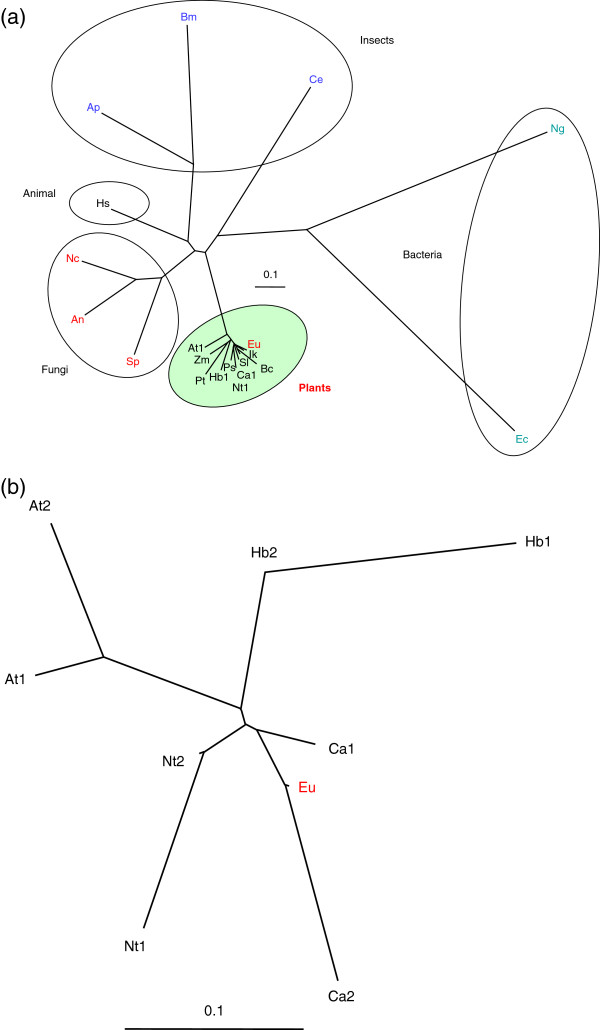
**Phylogenetic analysis based on comparison of deduced amino acid sequence of EuIPI with those of other IPIs from different organisms including plants, bacteria, fungi, and animals (a) or with plant two type I IPIs (b). **EuIPI shares a common evolutionary origin with other plant IPIs indicating that EuIPI belongs to the plant IPI group and has high homology with *C. acuminata* IPI2 (90.9% identity). Alignments were performed with Clustal W and visualized with TreeView. Distances between sequences are expressed as 0.1 changes per amino acid residue. Accession numbers and abbreviations: An, *Aspergillus nidulans* (AF479816); Ap, *Acyrthosiphon pisum* (FJ824667); At1, *Arabidopsis thaliana* (U47324); At2, *Arabidopsis thaliana* (U49259); Bc, *Bupleurum* chinense clone (GQ433719); Bm, *Bombyx mori* (AB274994), Ca1, *Camptotheca acuminata* (AF031079); Ca2, *Camptotheca acuminata* (AF031080); Ec, *Escherichia coli* (EU896065); Ce, *Caenorhabditis elegans* (AY597336); Eu, *Eucommia ulmoides* (AB041629); Hb1, *Hevea Brasiliensis* (AB294696); Hb2, *Hevea Brasiliensis* (AB294697); Hs, *Homo sapiens* (AF271720); Ik, *Ipomoea* sp. Kenyan (AB499048); Nc, *Neurospora crassa* (AB299023); Ng, *Natronobacterium gregoryi* (AJ564483); Nt1, *Nicotiana tabacum* (AB049815); Nt2, *Nicotiana tabacum* (AB049816); Ps, *Periploca sepium* (AB091677); Pt, *Pinus taeda* (GQ476784); Sl, *Solanum lycopersicum* (EU253957); Sp, *Schizosaccharomyces pombe* (U21154); and Zm, *Zea mays* (AF330034).

Phylogenetic analysis based on the comparison of the deduced amino acid sequence of EuIPI with those of other IPIs from different organisms including plants, bacteria, fungi, and animals demonstrated that EuIPI belonged to the plant IPI group (Figure 3a) and had high homology with *C. acuminata* IPI2 (90.9% identity, Figure 3b). The phylogenetic analysis suggested that all IPIs evolved from a common ancestor, and that EuIPI shared a common evolutionary origin with other plant IPIs.

### Analysis of EuIPI enzymatic activity

**Figure 4:**
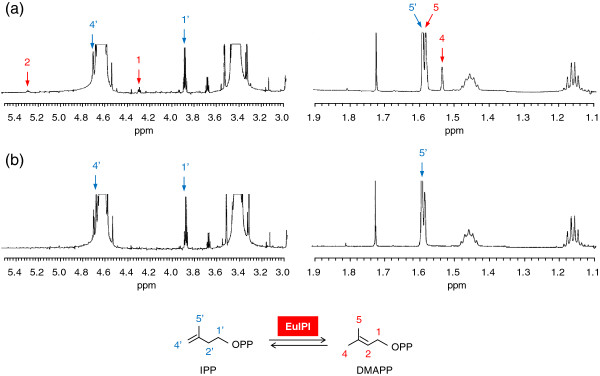
^**1**^**H-NMR spectra of reaction products obtained by incubating IPP substrate with (a) and without (b) purified EuIPI protein in enzymatic activity assay. **Red arrows indicate four signals characteristic of DMAPP (1.53, 1.58, 4.3, 5.3 ppm), which was produced from IPP catalyzed by EuIPI, and were absent from the reaction without EuIPI. Signals of IPP substrate (1.59, 2.3, 3.9, 4.7 ppm; blue arrows) were detected in the reaction either with or without purified EuIPI protein. These results indicated that the cDNA clone isolated from *E. ulmoides* encoded a functional IPP isomerase capable of catalyzing the conversion of IPP to DMAPP *in vitro.*

To confirm that the gene product EuIPI was a functional IPI, the cDNA was expressed in *Escherichia coli* BL21 via a pGEX-6P-1 expression system containing a glutathione S-transferase (GST)-tagged fusion protein sequence. This system is suitable for the production of soluble protein in the cytoplasm of *E. coli*. After induction with isopropyl thio-β-D-galactoside (IPTG), the *E. coli* cells harboring *EuIPI* produced a recombinant protein of approximately 56 kDa. The digested protein (≈26 kDa) was used for the EuIPI activity assay, in which its ability to catalyze the production of DMAPP from IPP was determined by measuring the amount of radioactivity incorporated into acid-labile reaction products (Table 1). The proton nuclear magnetic resonance (^1^H NMR) spectrum from the reaction mixture showed four signals characteristic of DMAPP (1.53, 1.58, 4.3, and 5.3 ppm; Figure 4a), which were absent from the reaction mixture without IPI protein (Figure 4b). The signals of the IPP substrate (1.59, 2.3, 3.9, 4.7 ppm; Figure 4a, 4b) were detected in the reaction mixture either with or without purified EuIPI protein. The spectra of IPP and DMAPP were nearly identical to those published previously [17]. These results unambiguously indicated that the digested EuIPI protein was able to catalyze the conversion of IPP to DMAPP *in vitro*, confirming that the cDNA clone isolated from *E. ulmoides* encoded a functional IPP isomerase.

**Table 1:** Analysis of EuIPI enzymatic activity

	**Background (no enzyme)**	**EuIPI (5 μg)**
[4-^14^C] IPP Incorporation (DPM)	65.92 ± 2.86	417.19 ± 4.86

For EuIPI enzymatic activity assay, the 50-μL reaction mixture contained 10 nmol [4-^14^C] IPP (37 GBq/mol) substrate and 5 μg purified protein (0.5 μg/μL). Radioactivity of reacted products was measured with a liquid scintillation counter as described in the Materials and methods. Distilled water (no enzyme) was used for background measurement; Data represent means ± standard error, *n*=3.

### Overexpression of *EuIPI* in *E. ulmoides* root lines

The cDNA of *EuIPI* was inserted into the pMSIsGFP vector, which was introduced into *E. ulmoides* roots via *Agrobacterium*-mediated transformation. Several kanamycin-resistant root lines with sGFP(S65T) (synthetic green-fluorescent protein with S65T mutation) fluorescence were obtained after selection and regeneration. PCR analysis confirmed that 25 root lines produced the predicted DNA fragment, indicating that the transgenes were present in these transgenic root lines. Some transgenic root lines showed defective phenotypes. Eight PCR-positive (PCR+) root lines showing strong growth were selected as representative *EuIPI*-overexpressing transgenic root lines for further analyses.

**Figure 5:**
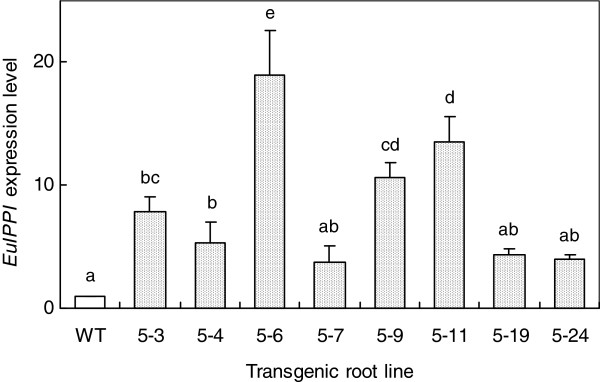
**Comparison of *EuIPI *expression levels between transgenic *E. ulmoides *root lines and wild-type control. ***EuIPI* was transformed into *E. ulmoides* via *Agrobacterium*-mediated transformation. Transgenic lines overexpressing *EuIPI* showed increased expression of *EuIPI* gene (19-fold higher expression in the pOEB5-6 line) compared with that in the wild-type control. Data represent means ± standard error, *n*=3; different letters indicate significant differences at *P* < 0.01 (ANOVA, Statistica, St. Tulsa, OK, USA).

Real-time RT-PCR analysis of the representative transgenic root lines showed that the expression level of the *EuIPI* gene was significantly increased in the overexpressing transgenic root lines (*P* < 0.01, Figure 5). The highest expression level was in pOEB5-6, which showed 19-fold greater expression than that of the endogenous gene in the wild-type (non-transgenic root line) control. These results confirmed that the overexpression approach successfully upregulated the expression level of *EuIPI* in transgenic *E. ulmoides* roots.

### *Trans*-polyisoprene analysis of transgenic *E. ulmoides* root lines

**Figure 6:**
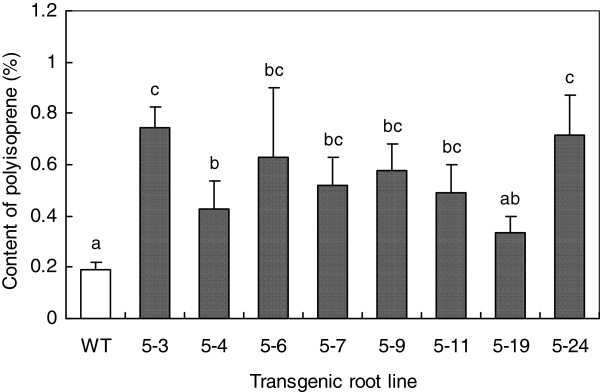
**Comparison of total *trans*-polyisoprenes contents between transgenic *E. ulmoides *root lines and wild-type control. ***EuIPI* was transformed into *E. ulmoides* via *Agrobacterium*-mediated transformation. Transgenic lines overexpressing *EuIPI* showed 3- to 4-fold increases in contents of *trans*-polyisoprenes compared with that in the wild-type control. Data represent means ± standard error, *n*=3; different letters indicate significant differences at *P* < 0.05 (ANOVA, Statistica).

The total contents of *trans*-polyisoprenes in the representative transgenic root lines and their molecular weight distribution were determined by pyrolysis-gas chromatography/mass spectrometry (PyGC/MS) and size exclusion chromatography (SEC). According to the report by Takeno et al. [18], the Soxhlet extraction method coupled with PyGC/MS analysis can remove low-molecular mass biosynthetic intermediates of *cis*- and *trans*-polyisoprenes, polyprenols, and other isoprenoids such as quinines (plastoquinone or ubiquinone), carotenoids, sterols, and chlorophylls. In addition, polyisoprenes isolated from bark and leaves of *E. ulmoides* via Soxhlet extraction and fractionation were confirmed to be pure *trans*-polyisoprene by ^13^C NMR [7]. Therefore, our PyGC/MS analysis was able to sensitively identify the *trans*-polyisoprene content in the transgenic lines of *E. ulmoides*. PyGC/MS analysis of eight representative *EuIPI*-overexpressing transgenic root lines showed that they contained increased total contents of *trans*-polyisoprenes (Figure 6), compared with the wild-type control. The *trans*-polyisoprenes contents in lines pOEB5-3, pOEB5-6, and pOEB5-24 were 3- to 4-fold greater than that in the wild-type control. An overall comparison by analysis of variance (ANOVA) showed that the total contents of *trans*-polyisoprenes in overexpressing transgenic root lines were significantly greater than those in the wild-type control (*P* < 0.05). These results indicated that up-regulation of *EuIPI* expression level increased the synthesis of *trans*-polyisoprenes in *E. ulmoides* root lines.

**Figure 7:**
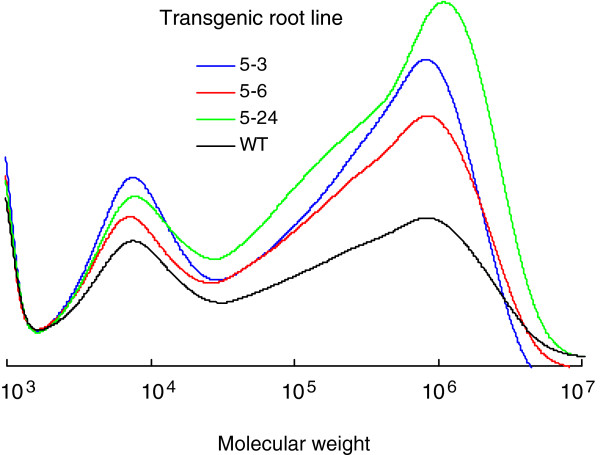
**Comparison of molecular weight distribution of *trans*-polyisoprenes between transgenic *E. ulmoides *root lines and wild-type control. **Compared with those in the wild-type control, the three representative transgenic lines overexpressing *EuIPI* showed significantly increased peak areas in both the low molar mass region from 1.5×10^3^ to 2.5×10^4^ and the high molar mass region from 2.5×10^4^ to 4.0×10^6^, especially the latter.

The molecular-weight distribution of the *trans*-polyisoprenes extracted from transgenic *E. ulmoides* root lines and the wild-type control showed a very broad (10^3^ to 10^7^) and distinct bimodal distribution pattern (Figure 7). The low- and high-molecular weight peaks were centered around 10^4^ and 10^6^, respectively. Compared with those in the wild-type control, the three representative root lines overexpressing *EuIPI* (pOEB5-3, pOEB5-6, and pOEB5-24) showed significantly increased peak areas in both the low molar mass region from 1.5×10^3^ to 2.5×10^4^ (*M*_*n*_=6.3×10^3^ and *M*_*w*_=8.0×10^3^) and the high molar mass region from 2.5×10^4^ to 4.0×10^6^ (*M*_*n*_=3.3×10^5^ and *M*_*w*_=9.3×10^5^), especially the latter. This increase was most evident in pOEB5-24. In the transgenic root lines pOEB5-3, pOEB5-6, and pOEB5-24, the low molar mass region accounted for 19.2, 16.1, and 13.3% of all *trans*-polyisoprenes, respectively, and the high molar mass region accounted for 80.7, 83.9, and 86.7%, respectively. In the wild-type control, the low molar mass region accounted for 26.2% of all *trans*-polyisoprenes and the high molar mass region accounted for 73.8%. These results indicated that the transgenic root lines produced higher molecular weight *trans*-polyisoprenes than did the wild-type control.

## Discussion

We aimed to up-regulate *IPI* expression to enhance *trans*-polyisoprene production in *E. ulmoides*. In the plant isoprenoid biosynthetic pathway (Figure 1), the basic five-carbon unit IPP is synthesized in the cytoplasm via the mevalonic acid (MVA) pathway [19,20] or in plastids via the 1-deoxy-D-xylulose-5-phosphate (DXP) pathway [9,19-22]. IPP is interconverted to its highly electrophilic isomer, DMAPP, by the enzyme IPI at the first step. Then, IPP is sequentially condensed to DMAPP to yield the short-chain isoprenoid precursors geranyl diphosphate (GPP), farnesyl diphosphate (FPP), and geranylgeranyl diphosphate (GGPP). These precursors are further metabolized for the biosynthesis of distinct sets of isoprenoids, such as monoterpenes (C10), sesquiterpenes (C15), diterpenes (C20), and polyisoprenes (C>5000) by various isoprenyl diphosphate synthases [20,23,24]. Therefore, IPP and DMAPP are starting materials at important regulatory branching points in the biosynthetic pathway of a variety of isoprenoids. IPI is thought to catalyze a regulatory step in isoprenoid biosynthesis [20]. It functions in supplying both the electrophilic primer substrate and the condensation substrate for isoprenoid biosynthesis, and provides the precursor for biosyntheses of various meroterpenoids [11,13,25].

According to previous studies, IPIs can be classified into two types: type I and type II. Type I IPIs have been identified in various eukaryotic organisms including humans [26] and *Saccharomyces cerevisiae*[19], and in some bacteria including *E. coli*[16] and *Rhodobacter capsulatus*[27]. The type II enzymes have been identified in archaea and some bacteria. These type II enzymes are FMN and NAD(P)H dependent [17]. To date, no type II IPI has been identified in the plant kingdom. Type I IPIs have a conserved C residue in a TNTCCSHPL motif and a conserved E residue in a WGEHEXDY motif [16]. Type II IPIs have a conserved motif that includes three G (glycine)-rich sequences, MTGG, GXGGT, and (A/G)SGG [17]. These highly conserved residues are critical for the catalytic activity of the enzyme [15,28-30]. Our phylogenetic analysis revealed that EuIPI shared a common evolutionary origin with other plant IPIs. EuIPI also contained the highly conserved C and E residues in the two motifs (Figure 2), suggesting that it belongs to the type I IPI family. The C and E residues are thought to face each other in the active site. The reaction is initiated by protonation of the double bond, a process that involves the E residue. The thiol moiety of C, presumably in the thiolate form, assists in removing the proton from the tertiary cation [29]. Most plants have two type I IPI isozymes with distinct subcellular localizations. In tobacco, IPIs are localized in the cytosol and plastids [31], while in castor bean, IPIs are present in mitochondria and proplastids. *Arabidopsis* also has two IPI genes that may function in the plastids [20,32,33]. Multiple alignment analysis showed that EuIPI shared high homology with *C. acuminata* IPI2 (Figure 2), a plastid IPI that resembles other plant IPIs [34]. At present, the subcellular localization of EuIPI is still unknown, and so further research is required to clarify its exact location within the cell.

Our results indicated that increasing the expression level of *EuIPI* increased synthesis of *trans*-polyisoprene in transgenic *E. ulmoides*. Overexpression of the *IPI* gene in *E. ulmoides* enhanced *trans*-polyisoprene production by 3- to 4-fold compared with that in the wild-type. Although the exact contribution of IPI to biosynthesis of *trans*-polyisoprene in transgenic *E. ulmoides* is unknown, our results demonstrated that regulation of *IPI* expression may be a key target for efficient production of *trans*-polyisoprene in *E. ulmoides*. A previous report on the prokaryote *E. coli* implied that maize IPI activity was critical for controlling the flux into the carotenoid pathway [35], and thus, represented an important step in isoprenoid biosynthesis. Similarly, in the green unicellular alga *Haematococcus pluvialis,* expressions of IPI and two enzymes specific to the carotenoid pathway (lycopene β-cyclase and β-carotene-C-4-oxygenase) resulted in a 3-to 6-fold increase in carotenoid accumulation after exposure to strong illumination [12]. On the other hand, silencing of the tobacco IPI led to a depletion of photosynthetic pigments, suggesting that reduced IPI activity affected isoprenoid biosynthesis in the plastids of tobacco leaves [36]. A site-directed specific inhibitor of IPI, 3,4-oxido-3-methyl-1-butyl diphosphate (OMBPP), inhibited incorporation of IPP into polyisoprene in an *in vitro* rubber assay [37]. Together, these findings imply that IPI catalyzes a key step in isoprenoid biosynthesis. Consequently, IPI is an attractive target for metabolic engineering for efficient production of industrially useful isoprenoids, including polyisoprene.

The transgenic root line pOEB5-6 showed the highest expression level of *EuIPI*, but did not show the highest accumulation of *trans*-polyisoprene. This may be because EuIPI is a rate-limiting enzyme [11,12,14,25,36] whose catalysis is down-regulated by feedback inhibition [38]. To increase accumulation of *trans*-polyisoprene via biochemical pathways, it may be necessary to manipulate regulatory genes such as kinases or transcription factors to up-regulate entire pathways [38].

Since a multi-branched metabolic pathway is responsible for the synthesis of distinct isoprenoids, overexpression of only the first-step enzyme, IPI, cannot enhance polyisoprene production to an extremely high level. Recently, we isolated a gene encoding *trans*-isoprenyl diphosphate synthase (EuTIDS, Accession No. AB041626) and its co-factors, which have roles in prenyl chain elongation and the formation of rubber particle proteins in *E. ulmoides* (data not shown). We anticipate that overexpression of these genes in addition to *EuIPI* will maximize the production of *trans*-polyisoprene in *E. ulmoides*.

*E. ulmoides* is a tertiary species that survives only in China [39], but it can be cultivated from tropical to temperate zones, and even in cold regions, whereas other species producing *trans*-polyisoprene (*M. balata* and *P. gutta*) grow only in the tropics. Hence, *E. ulmoides* shows greater promise as an industrial raw material for commercial use. The results of our study will be helpful to develop *E. ulmoides* for large-scale production of *trans*-polyisoprene by genetic engineering.

## Conclusions

To develop a plant-based system suitable for large-scale production of *trans*-polyisoprene, we selected a *trans*-polyisoprene-producing plant, *Eucommia ulmoides* Oliver, as the target for genetic transformation. A full-length cDNA (designated as *EuIPI*, Accession No. AB041629) encoding isopentenyl diphosphate isomerase (IPI) was isolated from *E. ulmoides*. *EuIPI* consists of 1028 bp with a 675-bp open reading frame encoding a protein with 224 amino acid residues. EuIPI shared high identity with other plant IPIs, and the recombinant protein expressed in *Escherichia coli* showed IPI enzymatic activity *in vitro*. *EuIPI* was introduced into *E. ulmoides* via *Agrobacterium*-mediated transformation. Transgenic lines of *E. ulmoides* overexpressing *EuIPI* showed increased *EuIPI* expression (up to 19-fold that of the wild-type) and a 3- to 4-fold increase in the total content of *trans*-polyisoprenes, compared with the wild-type (non-transgenic root line) control. IPI catalyzes the conversion of isopentenyl diphosphate to its highly electrophilic isomer, dimethylallyl diphosphate, which is the first step in the biosynthesis of all isoprenoids, including polyisoprene. Our results demonstrated that regulation of *IPI* expression is a key target for efficient production of *trans*-polyisoprene.

## Methods

### cDNA library construction

Total RNA was extracted from leaves of a mature *E*. *ulmoides* tree using the cetyltrimethylammonium bromide (CTAB) method [40]. The mRNA was purified from total RNA using Oligotex-dT30 Super (Takara Bio, Otsu, Shiga, Japan) and was used to construct a cDNA library using a lambda ZAP II XR Library Construction kit (Stratagene Japan, Tokyo, Japan).

### Cloning of full-length *EuIPI* cDNA

The total RNA was reverse-transcribed to synthesize first-strand cDNA using an AMV Reverse Transcriptase XL kit and Oligo dT adaptor primers (Takara Bio). To amplify the *EuIPI* cDNA fragment, one pair of degenerate primers (forward: 5^′^- TTI GTI TGG ACI AAY ACN TGY TG-3^′^ and reverse: 5^′^-AAA IAG IAG RTA RTC IAN YTC ATG YTC-3^′^, where N is A, C, G or T; Y is C or T; R is A or G, and I is inosine) was designed according to a region that is highly conserved among known plant IPIs (Figure 3). The PCR conditions were as follows: 5 min at 94°C for preheating, 30 cycles of 1 min at 94°C for denaturation, 1 min at 54°C for annealing, 2 min at 74°C for synthesis, and 7 min at 74°C for final extension. The PCR product was labeled using the AlkPhos Direct Labeling and Detection System with CDP-STAR (GE Healthcare Japan, Tokyo, Japan) and used as a probe to screen the cDNA library. Phage plaques were lifted onto a Hybond N+ membrane (GE Healthcare Japan) and hybridized with the labeled probe under the conditions specified by the manufacturer. The positive lambda ZAP II clones were excised *in vivo* with helper phage to generate subclones in the pBluescript SK(−) phagemid vector (using a lambda ZAP II XR Library Construction kit; Stratagene Japan) and transformed into *E. coli* SOLR cells (Stratagene Japan) for sequencing. One clone carrying a full-length cDNA insert was chosen and designated as *EuIPI*.

### Phylogenetic analysis

The deduced amino acid sequence of EuIPI was aligned against those of other IPIs from different organisms, including plants, bacteria, fungi, and animals, by the Clustal W method with default parameters (Slow-Accurate) using Lasergene® MegAlign (DNASTAR, Madison, USA). A phylogenetic tree was constructed by using the neighbor-joining method [41] using Treeview (Glasgow, Scotland, UK).

### Analysis of EuIPI enzymatic activity

The *EuIPI* cDNA, spanning from the start codon (ATG) to the stop codon (TAG) (Figure 2), was amplified by PCR with the following primers: forward: 5^′^-TAT CTC GAG ATG GGT GAT ACC GCC GTC-3^′^ (*Xho*I site underlined) and reverse: 5^′^-TAT GCG GCC GCT AAG CAG ACT GAT TTT C-3^′^ (*Not*I site underlined). The PCR product was inserted into a *Xho*I- and *Not*I-digested pGEX-6P-1 vector (GE Healthcare Japan) to yield an expression plasmid with a GST-tagged fusion protein sequence. The plasmid was introduced into *E. coli* BL21. The recombinant proteins were harvested from the *E. coli* transformant cells after being induced by addition of 0.2 mM IPTG. The GST fusion protein was purified by affinity chromatography using a GSTrap FF column (GE Healthcare Japan), and the GST-tag was removed by enzymatic cleavage using PreScission Protease (GE Healthcare Japan). The purified protein was used for the EuIPI activity assay and ^1^H NMR analysis according to the methods described by Kaneda et al. [17] with some modifications according to the optimum conditions for enzyme activity. For the assay, 10 nmol [4-^14^C] IPP (37 GBq/mol) (PerkinElmer Japan, Yokohama, Japan) substrate and 5 μg purified protein (0.5 μg/μL) were incubated in a 50-μL reaction mixture containing 100 mM Tris–HCl (pH 7.0), 5 mM MgCl_2_, 25 mM NaF, and 1 mM dithiothreitol at 30°C for 20 min. The reaction was terminated by adding 200 μL methano1:HCl (4:1, v/v) and 500 μL water. Lactonization of samples was then carried out at 37°C for 10 min. The incubation mixture was saturated with NaCl and the allylic products were extracted twice with 500 μL toluene. The supernatant toluene layer was collected and dried over Na_2_SO_4_. The toluene phase (500 μL) was mixed with 3 mL cocktail and the radioactivity of reacted products was measured with a Tri-Carb 2100 liquid scintillation counter (Packard Instrument, Connecticut, USA).

The purified protein (1 mg) was also reacted with unlabelled 5 mM IPP (Sigma-Aldrich, St. Louis, MO, USA) in a 1649-μL reaction mixture containing 100 mM Tris–HCl (pH 8.5), 5 mM MgCl_2_, 25 mM NaF, and 1 mM dithiothreitol at 30°C for 16 h. After incubation, the reaction mixture was lyophilized, and the resulting residue was resuspended in 99.9% D_2_O. The reaction products were then analyzed using a 500 MHz ^1^H NMR spectrometer (Agilent Technologies Japan, Tokyo, Japan). As a control, a reaction mixture that did not contain purified protein was also analyzed.

### Construction of plant overexpression vector

**Figure 8:**
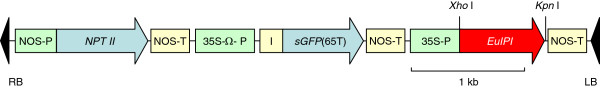
**Schematic structure of T-DNA region of overexpression vector used for *E. ulmoides *transformation. **cDNA of *EuIPI* was inserted into pMSIsGFP binary vector between 35S promoter and a NOS terminator. *NPT II* gene was used as a selective marker, and *sGFP(S65T)* gene was used to optimize conditions for transformation by monitoring the expression of green-fluorescent protein. An intron was fused within the N-terminal part of the sGFP(S65T) coding sequence to discriminate between *Agrobacterium* and plant expression (because bacteria can not splice the intron). RB, right border; LB, left border; NOS-P, nopaline synthase promoter; NOS-T, nopaline synthase terminator; 35S-P, cauliflower mosaic virus (CaMV) 35S promoter; 35S-Ω-P, 35S promoter with additional omega element translational enhancer; NPT II, neomycin phosphotransferase; sGFP(65T), synthetic green-fluorescent protein with S65T mutation; I: intron of castor bean catalase gene *CAT-1.*

The fragment of *EuIPI,* spanning from the start codon to the stop codon (Figure 2), was amplified by PCR using the following primers: forward: 5^′^-TAT CTC GAG ATG GGT GAT ACC GCC GTC-3^′^ (*Xho*I site underlined) and reverse: 5^′^-GCT GGT ACC CTA AGC AGA CTG ATT TTC C-3^′^ (*Kpn*I site underlined). The PCR product was directionally inserted into the *Xho*I and *Kpn*I sites of a pMSIsGFP binary vector between a cauliflower mosaic virus (CaMV) 35S promoter and a nopaline synthase (NOS) terminator to construct the overexpression vector pOEB5 (Figure 8). The pMSIsGFP vector was modified from a pMSH1 vector [42] by introducing a cassette containing a 35S-Ω promoter (35S promoter with additional omega element translational enhancer [43])-driven *sGFP(S65T)* gene [44] and a NOS terminator from pBIsGFP(S65T) [45] inserted at the *Hin*dIII sites. An intron from the castor bean catalase gene *CAT-1* was fused within the N-terminal part of the sGFP(S65T) coding sequence to discriminate between *Agrobacterium* and plant expression (because bacteria cannot splice the intron). The resultant vector pOEB5 was introduced into *A. tumefaciens* strain LBA4404 [46] using a freeze-thaw transformation method [47].

### Overexpression of *EuIPI* gene in *E. ulmoides* root lines

For gene transformation, we used a proliferated root line from a 4-week-old germfree seedling of *E. ulmoides*. The root line was kept in suspension culture in a root proliferation liquid medium containing half-strength Murashige and Skoog (MS) basal medium (half-strength MS salts and vitamins), supplemented with 15 g/L sucrose and 1 μM naphthaleneacetic acid (NAA). The *A. tumefaciens* strain LBA4404 harboring the pOEB5 vector was grown overnight at 28°C with shaking (150 rpm) in Luria-Bertani liquid medium containing 50 mg/L kanamycin. Bacterial cells were collected by centrifugation and resuspended to a final OD_550_ of 0.25 in suspension solution containing MS basal medium supplemented with 30 g/L sucrose, 3 μM 6-benzylaminopurine (BAP), and 3 μM 6-(γ,γ-dimethylallyl-amino)purine (2-iP) combined with 20 mg/L acetosyringone. The proliferated clonal roots of *E. ulmoides* were cut into 5–8 mm segments, sonicated for 20 min, and immersed in *Agrobacterium* suspensions for 3 min. Segments were then blotted dry with sterile filter paper to remove excess bacteria, and transferred into Petri dishes containing filter paper laid over co-cultivation medium (same composition as suspension solution, but solidified with 2.4 g/L Gelrite (Wako Pure Chemical Industries, Osaka, Japan). After 3 days of co-cultivation at 22°C in the dark, segments were transferred to callus induction and selection medium (same composition as the co-cultivation medium, but with no acetosyringone, and containing 200 mg/L vancomycin and 25 mg/L kanamycin). They were subcultured twice over a 3-week interval, and then transferred to root induction medium (same composition as root proliferation liquid medium, but solidified with 2.4 g/L Gelrite, and containing 200 mg/L vancomycin and 25 mg/L kanamycin). After the calli regenerated adventitious roots, only one well-grown root from each transgenic callus was harvested and transferred to the root proliferation liquid medium containing 200 mg/L vancomycin and 5 mg/L kanamycin for proliferation. The roots proliferated from a transgenic callus were considered as an independent root line. All cultures were incubated at 25°C under a 16-h light/8-h dark photoperiod with light supplied by a fluorescent cool-white light (50 μmol m^-2^s^-1^ photosynthetic photon flux density).

### DNA and RNA analyses of transgenic roots

PCR analysis was conducted to screen transgenic roots for the presence of the transgenes. Total genomic DNA was isolated from the regenerated root lines using the DNeasy Plant Mini kit (Qiagen K.K., Tokyo, Japan) according to the manufacturer’s instructions. To distinguish the endogenous *EuIPI* from the transgene, a pair of primers (forward: 5’-TCA TTT GGA GAG AAC ACG GGG GAC-3’ and reverse: 5’-TGC TCT TGG ACG TTG CAA ACG TAAG-3’) was designed based on the T-DNA sequence of pOEB5 (one from the sequence of the 35S promoter and one from that of the NOS terminator, located either side of the *EuIPI* cDNA overexpression construct). These primers were used to amplify the 820-bp fragment by PCR using 20 ng total isolated DNA as the template. The PCR conditions were as follows: 5 min at 95°C for preheating, 30 cycles of 1 min at 95°C for denaturation, 1 min at 60°C for annealing, 2 min at 74°C for synthesis, and 7 min at 74°C for final extension. Eight PCR+ root lines showing strong growth were selected as the representative *EuIPI*-overexpressing transgenic root lines for further analyses.

Total RNA was isolated from the eight representative transgenic root lines using an RNeasy Plant Mini kit (Qiagen K. K, Tokyo, Japan). The expression level of *EuIPI* in these lines was determined by real-time RT-PCR as described previously [48]. The primers used for RT-PCR analysis of the *EuIPI* gene were as follows: 5^′^-AAC GAT CAG GGA CAA AGG TAA CA-3^′^ (forward) and 5^′^-GGA TGG CTG CAG CAT GTG-3^′^ (reverse). Gene expression was calibrated against that of an endogenous gene, elongation factor-1 alpha (*EF1α*), which was amplified using the primers 5^′^-CCG AGC GTG AAC GTG GTA T-3^′^ (forward) and 5^′^-TAG TAC TTG GTG GTT TCG AAT TTC C-3^′^ (reverse).

### Analysis of *trans*-polyisoprenes in transgenic roots

The total content of *trans*-polyisoprenes in each transgenic root line and the distribution of their molecular weights (about 10^2^–10^8^ M) were determined using PyGC/MS and SEC, according to our previous reports [18]. The Soxhlet extraction method was used before PyGC/MS and SEC analyses. Briefly, the sample was lyophilized and ground into a fine powder (150 mg) and then successively extracted by the Soxhlet method with ethanol (100 mL) at 120°C for 10 h and toluene (50 mL) at 150°C for 12 h. The residue obtained from toluene extraction was rinsed with methanol. The residue was dried with a centrifugal concentrator and then dissolved in toluene. After centrifugation, the supernatant was collected as *trans*-polyisoprene fractions for PyGC/MS and SEC analyses. For PyGC/MS analysis, polybutadiene rubber (BR, 1 mg) was added as an internal standard. To quantify *trans*-polyisoprene, the ratio of the area of *trans*-polyisoprene to that of BR was compared using NIST MS Search 2.0 (Agilent Technologies, Santa Clara CA, USA). For SEC analysis, a calibration curve was generated using 1,4-polyisoprene standards with SIC-480II GPC software (System Instruments, Tokyo, Japan). The molecular weight distribution was bimodal, and the relative areas of low- and high-molecular weight compounds were calculated.

## Competing interests

The authors declare that they have no competing interests.

## Authors’ contributions

RC drafted the manuscript, conducted molecular cloning, carried out functional and phylogenetic analyses of EuIPI, constructed vectors, analyzed overexpression of *EuIPI* in *E. ulmoides*, regenerated transgenic plants, and conducted DNA/RNA analyses. YH analyzed *trans*-polyisoprene in transgenic lines. TB constructed the cDNA library from *E. ulmoides*, carried out molecular cloning, analyzed enzymatic activity of EuIPI, developed the analysis of *trans*-polyisoprene in transgenic lines, and helped draft the manuscript. YN conceived the study and participated in its design and coordination. KG participated in the design and coordination of the study. All authors read and approved the final manuscript.
